# WDCN: a comprehensive neural network based approach for estimating breast cancer risk

**DOI:** 10.1093/bib/bbag412

**Published:** 2026-08-06

**Authors:** Hanshi Xu, Guangquan Zhang, Hua Lin, Mark Grosser, Jie Lu

**Affiliations:** Australian AI institute, Faculty of Engineering and Information Technology, University of Technology Sydney, 61 Broadway, Sydney 2007, New South Wales, Australia; Australian AI institute, Faculty of Engineering and Information Technology, University of Technology Sydney, 61 Broadway, Sydney 2007, New South Wales, Australia; 23Strands, 26-32 Pirrama Road, Pyrmont 2009, New South Wales, Australia; 23Strands, 26-32 Pirrama Road, Pyrmont 2009, New South Wales, Australia; Australian AI institute, Faculty of Engineering and Information Technology, University of Technology Sydney, 61 Broadway, Sydney 2007, New South Wales, Australia

**Keywords:** breast cancer, artificial neural network, bioinformatics approach

## Abstract

Breast cancer is one of the most distressing cancers affecting women, and early detection is considered the most effective way to reduce breast cancer mortality. However, the benefits of early detection vary among different risk groups. Therefore, using a combination of genetic information, family history, and other factors to stratify populations by risk can help more people benefit from early detection. Traditional polygenic risk score (PRS) is essentially a weighted sum calculation method that has achieved some success, but it neglects the interactions between genes–genes, genes–environment, and their potential impact on breast cancer risk. In this context, we developed a new deep learning-based method called wide, deep, and cross network (WDCN). Experimental results show that our algorithm outperforms PRS and other machine learning baseline methods and achieves an area under the receiver operating characteristic curve (AUROC) of 0.6439 when using 286 single nucleotide polymorphism (SNP) features and 0.8865 when incorporating environmental features with genetic data. Increasing the SNP set to 317 further raised the performance to 0.6464 and 0.8872, both with and without non-genetic factors. Risk stratification shows that individuals in the top 30% have a relative risk of 7.85 (95% CI: 6.98–8.83) compared with those in the bottom 30%. We also identified an interaction between rs2588809 and age. This novel approach has shown promise for initial risk stratification of populations, potentially providing better decision-making support for individuals and clinicians.

## Introduction

Worldwide, breast cancer is prominently recognized as the most diagnosed malignant neoplasm in females and among the deadliest forms of cancer. According to data from 2022, there were roughly 2.3 million instances of newly diagnosed breast cancer, representing $\sim $11.6% of the total global cancer incidence rate [1, 2]. The number of deaths from breast cancer accounts for $\sim $6.9% of all cancer-related deaths, ranking fourth in mortality [2]. Despite varying standards and methods for grading cancers in different countries, breast cancer treatment exhibits a consistent trend. Early stage (stage I, stage II, stage III, or localized and regional stage) breast cancers have an 80% or higher 5 year survival rate [3–7], while advanced (stage IV or distant) breast cancers have only a roughly 40%–20% survival rate [3–7]. The significant decrease in survival rates suggests the importance of early diagnosis and treatment, with some studies [8, 9] also confirming that early detection can indeed improve survival rates.

Mammography is the most widely used detection method in breast cancer [10], as it balances accuracy with cost. Many clinical guidelines [11, 12] recommend that women over 40 or 50 years old should undergo annual or biennial mammography for breast cancer screening. However, not everyone benefits from it. For individuals at high risk of breast cancer, mammography may lead to false negatives, and the more sensitive magnetic resonance imaging (MRI) is considered a more suitable screening method [13]. For individuals at low risk, mammography carries the risk of overdiagnosis, leading to unnecessary anxiety and radiation exposure [14, 15]. Thus, different risk groups should be recommended different screening strategies, which may bring greater benefits to individuals [16].

Polygenic risk score (PRS) is a commonly used method for risk stratification [17, 18]. The PRS is essentially a weighted score, where weights are assigned to each genetic variant based on its association with breast cancer in case-control studies. These weights are then used to calculate the individual's risk of recurrence at each genomic location, and finally, the scores from all selected loci are averaged to obtain the overall risk score.

This method appears straightforward and easy to interpret, but it overlooks the collinearity between SNPs and consequently loses a substantial amount of information. To address these limitations, several improved PRS methods have been developed [19, 20]. These methods calculate the collinearity or linkage disequilibrium (LD) relationships between different SNPs, recalculate the weights of individual SNPs. However, both traditional and improved PRS methods rely on the additive aggregation of SNP effects, overlooking the widespread gene–gene and gene–environment interactions, and therefore are considered insufficient for accurately assessing disease risk [21].

Random Forest (RF) [22] and XGBoost [22–24] methods have also been applied to breast cancer risk estimation, where these machine learning algorithms learn the contribution of each SNP. While not as sophisticated as some other approaches that incorporate LD correction, these methods do take into account the interactions between SNPs to some extent. In RF and XGBoost, the contributions assigned to each SNP are determined by how well each SNP or SNP combination can predict breast cancer risk. These approaches are capable of capturing interactions between features and allow the algorithm to identify the most informative SNPs and SNP combinations for predicting breast cancer risk.

Wide and deep network (WAD) [25] and deep and cross network (DCN) [26, 27] are algorithms invented by Google, leveraging deep learning to implicitly find interactions between features. WAD combines a wide part and a deep learning part. The wide part can capture the linear weights of each feature, while the deep learning part uses fully connected neural networks to capture the non-linear importance of features. DCN does not have a wide part, instead directly using feature interactions, iteratively reducing the residual, and then using fully connected neural networks to model and predict results. These two deep learning algorithms are mainly used for applications such as advertisement recommendation. In genetic studies, various deep learning methods have also been introduced to evaluate the risk of complex diseases such as breast cancer [28] and cardiovascular disease [29]. These complex diseases involve numerous interactions [30, 31], including epistatic relationships among genes [32, 33]. Deep learning methods are capable of capturing this complexity and therefore can provide more accurate risk assessments [34, 35].

In this study, we first performed a genome-wide association study (GWAS) analysis and verified 21 loci associated with breast cancer risk. Although our study population consisted exclusively of individuals of European ancestry, subsequent linkage analysis suggested the presence of potential subpopulation genetic structures. Then we developed a new neural network architecture: wide, deep, and cross network (WDCN) that incorporates both simple linear additive components and feature interaction parts. This design allows us to fully capture the characteristics of individual features while also capturing interactions between different SNP features. We trained and tested our model using two datasets: one consisting of 286 SNP features and another consisting of 317 SNP features in samples from the UK Biobank database (UKBB). We used the same dataset as before, with the addition of non-genetic features. In all four testing scenarios, our model performed well, outperforming traditional models in terms of AUROC. Furthermore, we found that the risk of developing breast cancer among individuals in the high group was 7.85 times higher than those in the low-risk group. SHapley Additive exPlanations (SHAP) analysis was performed to evaluate and rank the importance of all features. Notably, changes in feature importance rankings after the addition of the non-genetic features revealed a potential interaction between rs2588809 and age. Our model demonstrated strong performance and showed potential to improve population classification, providing better decision-making references for clinicians and individuals.

## Material and methods

This section describes the overall design of our study, data acquisition, data processing, and model structure.

### Overall design of our study

**Figure 1:**
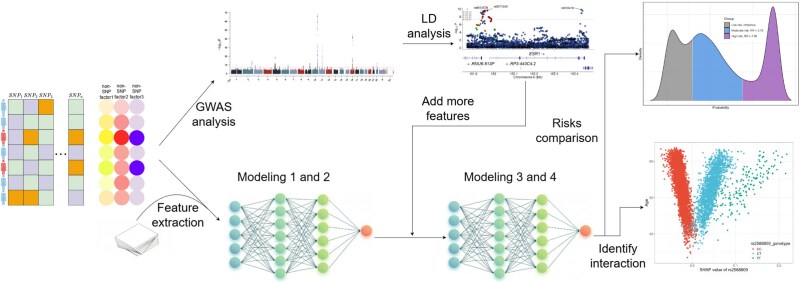
Overall design of our study, where UK Biobank data underwent quality control, genetic and non-genetic risk factors were integrated to develop four predictive models, GWAS and LD analyses were performed to characterize population genetic backgrounds, and risk changes were evaluated across different stratifications using the final model

Our overall experimental design is illustrated in Fig. 1. Firstly, we filtered the data from UK Biobank (UKBB), including sample information and genetic information, to obtain high-quality data. Subsequently, we conducted GWAS analysis and LD analysis, which revealed potential population substructures and various mechanisms underlying breast cancer in our cohort. We retrieved widely accepted SNPs and non-genetic factors associated with breast cancer risk from the literature. Then, we extracted this information from UKBB to test the performance of the baseline model and our proposed model. Afterward, we analyzed the possible reasons for the differences in model effectiveness. Due to the possibility of different population genetic structures and diverse breast cancer mechanisms, we re-extracted independent, significant loci from the GWAS and LD analyses, and constructed a new model. In the new model, we categorized individuals into different risk groups and compared the risk enhancement of different groups relative to a low-risk reference group.

### Data processing

All the data we used was from UKBB. UKBB [36, 37], a vast biobank, houses not only whole genome chip data but also individual health-related information. It is an extensive biomedical database that gathers genomic and health-related data from participants to enhance our understanding of the genetic and biological underpinnings of common diseases, ultimately aiming to promote preventive strategies and treatment approaches in research.

UKBB imputed Chip data was uesd for GWAS analysis, and the model was developed. Individuals were rigorously selected according to the information provided by UKBB. As this study focuses on female breast cancer, male individuals were initially excluded. Those individuals who did not have breast cancer were initially grouped into the non-breast cancer cohort, and those diagnosed with breast cancer were initially selected out into the breast cancer cohort. For non-breast cancer cohort individuals who have been diagnosed with any other types of cancer, benign tumors, and those who underwent mastectomy were also excluded. For the breast cancer cohort, individuals who were diagnosed with another type of cancer within 5 years of being diagnosed with breast cancer were also removed. After combining both cohorts, a gender check was performed. The degree of sex chromosome heterozygosity was used to determine if it matched the individual's self-reported gender. All individuals with a discrepancy between their self-reported gender and genetic sex were excluded. At last, the individuals who claimed to be of European descent were preserved.

The genetic feature or SNP data were initially subjected to quality control. All ambiguous bases, which were with a minor allele frequency (MAF) between 0.4 and 0.5 and consisted solely of C and G or A and T, were removed. All samples with an SNP calling rate under 0.90 were removed. SNPs that were called in more than 90% of samples were kept. After that, SNPs with MAF less than 0.001 were removed. Hardy–Weinberg equilibrium test was used to remove the SNPs that deviate from equilibrium under the thresholds of $10^{-5}$.

Subsequently, non-SNP features were extracted. These features included BMI, weekly alcohol consumption, smoking history, family history of breast or prostate cancer, current age, age at death (if deceased), age at breast cancer diagnosis (for breast cancer cases), and age at menopause. For BMI, weekly alcohol consumption, smoking history, family history of breast or prostate cancer, and age at menopause, all missing values were imputed using the mean of the non-missing values. For individuals with breast cancer, age was defined as the youngest among current age, age at breast cancer diagnosis, and age at death. For individuals without breast cancer, age was defined as the younger of current age and age at death. All subsequent age-related features were based on this definition. Years since menopause was defined as age minus age at menopause. Positive values indicate that menopause has already occurred, with higher values representing a longer time since menopause. Negative values indicate that menopause has not yet occurred, with lower values corresponding to a greater time remaining until the expected age at menopause.

### GWAS analysis and training test dataset splitting

GWAS was first performed on the entire dataset by plink2.0 [38], with the top ten PCs and age as covariates. Due to data imbalance, with the number of non-breast cancer individuals being $\sim $10.5 times that of breast cancer cases, we randomly assigned around 80% of breast cancer cases to the training set and the remaining 20% to the test set. Then, in the test set, we randomly selected an equal number of non-breast cancer individuals to match the number of breast cancer cases, while all remaining non-breast cancer individuals were included in the training set. This resulted in an imbalanced training set with a case to control ratio of $\sim $1:13 and a balanced test set with a 1:1 ratio. The demographic characteristics of the training set, test set, and overall study population are summarized in Table 1.

**Table 1:** Demographic information of the study population.

**Dataset**	**Class^a^**	**Numbers**	**BMI**	**Alcohol consumption**	**Age**	**Years to menopause**	**Ever smoke**	**Family history**
Entire	0	180 906	$27.0 \pm 5.1 $	$2.9 \pm 1.5 $	$72.1 \pm 7.9 $	$22.3 \pm 8.6 $	101 733 (56.2%)	32317 (17.9%)
dateset	1	17 088	$27.3 \pm 5.0$	$2.9 \pm 1.5 $	$59.0 \pm 9.6 $	$9.4 \pm 9.8 $	10 108 (59.2%)	4355 (25.5%)
Training	0	177 488	$27.0 \pm 5.1 $	$2.9 \pm 1.5 $	$72.1 \pm 7.9 $	$22.3 \pm 8.6 $	99 841 (56.2%)	31 687 (18.0%)
dateset	1	13 670	$27.3 \pm 5.0 $	$2.9 \pm 1.5 $	$59.0 \pm 9.6 $	$9.3 \pm 9.8 $	8098 (59.2%)	3450 (25.2%)
Testing	0	3418	$27.0 \pm 5.3 $	$2.9 \pm 1.5 $	$72.0 \pm 7.9 $	$22.2 \pm 8.6 $	1919 (56.1%)	620 (18.1%)
dataset	1	3418	$27.3 \pm 4.9 $	$2.9 \pm 1.5 $	$59.0 \pm 9.6 $	$9.4 \pm 9.7 $	2010 (58.8%)	905 (26.5%)

Continuous variables, such as BMI, are presented as mean $\pm $ SD, while categorical variables, such as ever smoke and family history, are presented as the number and percentage of individuals with the respective characteristic.

^a^Within each class, 0 indicates non-breast cancer cohort and 1 indicates breast cancer cohort.

### WDCN model

**Figure 2:**
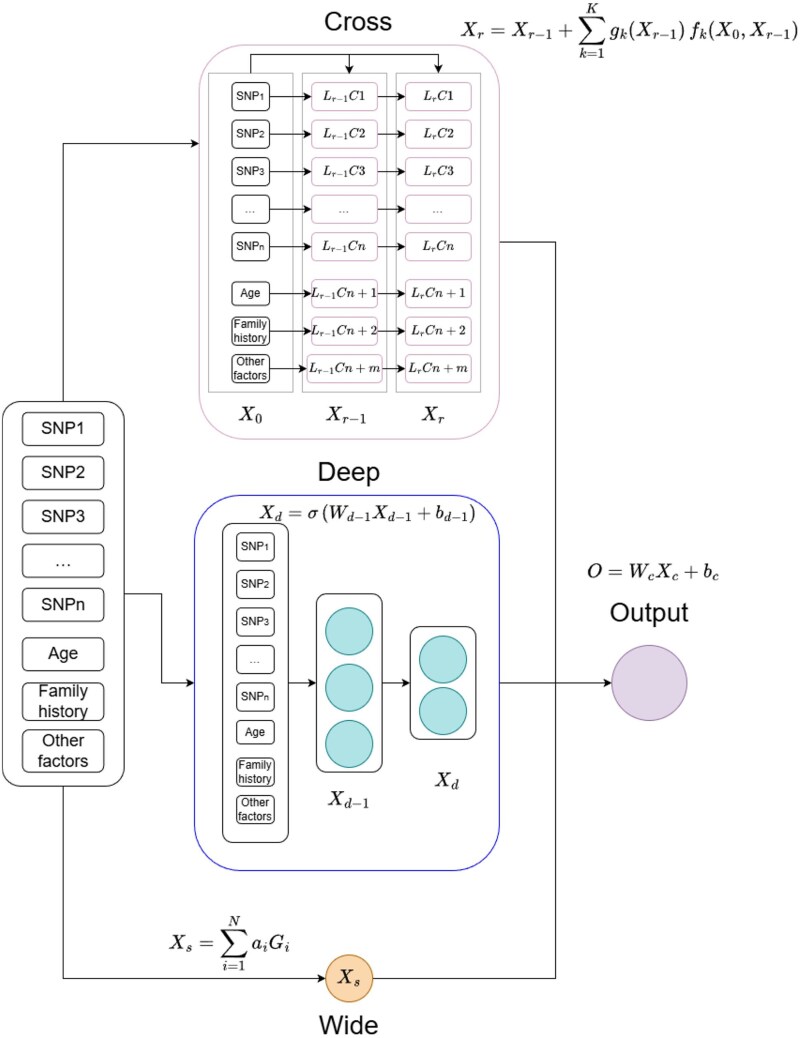
The WDCN model integrates three parallel components to capture linear, nonlinear, and interaction effects of features, whose outputs are combined to generate the final prediction.

The WDCN model architecture is illustrated in Fig. 2. This model mainly consists of three parallel parts: the wide part, the deep neural network part, and the cross part. All features are processed through these three components. The wide part is to calculate the weighted sum of each component. Since the entire process is to find the best fit in training, no prior consideration of independence between SNPs is necessary. The deep neural network part is a fully connected neural network, which aims to learn the non-linear relationships among the features. The cross part is designed to learn the interactions among features. Finally, a merge layer integrates the outputs of all three components and generates the prediction.

The formula for the wide part is: 


\begin{align*} &X_{s}=\sum_{i=1}^{N}a_iG_i\end{align*}

where $X_{s}$ is the final output of the linear part, $G_{i}$ is the $i$th feature, $a_{i}$ is the weight of $i$th feature and $N$ is the total number of features.

For the deep network part, the calculation formula is as follows: 


\begin{align*} &X_d=\sigma\left(W_{d-1}X_{d-1}+b_{d-1}\right)\end{align*}

Where $X_{d}$ is the $d$th layer of the deep network, $\sigma $ is the activate function. In this study, we use gaussian error linear unit (GELU) as the activate function. $W_{d}$ is the weight of $d$th layer, $b_{d-1}$ is bias.

In the cross part, we introduce a mixture-of-experts mechanism, allowing different experts to learn distinct patterns of feature interactions. An entire formula is as follows: 


\begin{align*} &X_r = X_{r-1} + \sum_{k=1}^{K} g_k(X_{r-1}) \, f_k(X_0, X_{r-1})\end{align*}

Here:

-
$X_{r} \in \mathbb{R}^{d}$ is the input to the $r$th cross layer.
-
$X_{0} \in \mathbb{R}^{d}$ is the original input.
-
$K$ is the number of experts.
-
$f_{k}(X_{0}, X_{r-1}) \in \mathbb{R}^{d}$ is the output of the $k$th expert.
-
$g_{k}(X_{r-1})$ is the gating weight for the $k$th expert.

This residual formulation ensures stable gradient propagation and allows the network to capture high order interactions. Each expert learns a distinct pattern of feature interactions. For a given expert, the corresponding function is: 


\begin{align*} &f(X_0, X_{r-1}) = X_0 \odot (T(W_{r-1}) X_{r-1} + b_{r-1})\end{align*}

where $T(W_{r-1})$ is a two step filter function to remove self interactions and control noise. 


\begin{align*} &T(W_{r-1}) = W_{r-1} \odot D \odot \mathbf{1}_{[|W_{r-1}|> \tau]}\end{align*}

Here:

-
$W_{r-1},D,I \in \mathbb{R}^{d \times d}$ .
-
$\odot $ denotes element wise multiplication.
-
$D$ is a matrix with 0 on the diagonal and 1 elsewhere to remove self interactions.
- Elements of $I$ are 1 where $|W_{r-1}|> \tau $ and 0 otherwise.
-
$\tau $ is the threshold.

To reduce the number of parameters and increase efficiency, we adopt a low-rank representation of the interaction weights: 


\begin{align*} &W_{r-1} \approx U_{l} \times V_{l}^T \end{align*}

where $U,V \in \mathbb{R}^{d \times l}$, $l \ll d$, $l$ is the low rank number.

The outputs of all experts are combined using a gating network: 


\begin{align*} &g(X_r) = \operatorname{softmax}(f_k(X_0, X_r))\end{align*}

The last part is combination part, which is combined the linear part and the deep cross part. The formula is as follows: 


\begin{align*} &O=W_cX_c+b_c\end{align*}

where $W_{c}$ is the weight of combined layer, $X_{c}$ is combined one with result of linear part and deep cross part. $b_{c}$ is the bias.

For loss function, we use weighted loss function to address data imbalance issue: 


\begin{align*} &L(\mathbf{w}) = - \left[ w_1\, y \log(p) + w_0\, (1-y)\log(1-p) \right]\end{align*}

where $L(\mathbf{w})$ is the loss function, $w_{1}$ is the weight of the minority class, $w_{0}$ is the weight of the majority class, $y$ is the true label and $p$ is the probability of the positive class predicted by the model.

We also introduce the L2 regularization method to reduce the risk of overfitting: 


\begin{align*} &\tilde{L}(\mathbf{w}) = L(\mathbf{w}) + \frac{\lambda}{2} \|\mathbf{w}\|_2^2\end{align*}

where $\mathbf{w}$ is all the parameters and $\lambda $ is L2 regularization parameter.

### Result normalization

To fairly compare the results of different methods, especially PRS, whose scores often do not fall within the 0–1 range, we applied the min–max normalization method to the results of all methods. 


\begin{align*} &X^{\prime}_{ij} = \frac{X_{ij} - \min(X_j)}{\max(X_j) - \min(X_j)}\end{align*}

where $X^{\prime}_{ij}$ is the normalized result for $i$th sample in $j$th method, $X{ij}$ is the original result.

### Hyperparameter tuning strategies and computational resources

We manually tuned the parameters via a one-factor-at-a-time approach. We initially assigned empirical values to all hyperparameters, then tuned the batch size and learning rate until the performance was optimized. After fixing these two parameters, we sequentially tuned the remaining hyperparameters until all had been adjusted. The specific tuning range for each hyperparameter, as well as the final parameter values used in the model, are presented in Supplementary Table S1.

We also used early stopping, learning rate decay, and similar techniques to avoid overfitting. The plots of model loss reduction are presented in Supplementary Figs S2–S4.

All experiments were conducted on a single NVIDIA L4 GPU with 24 GB of memory. The software environment consisted of PyTorch 2.4.0+cu121, CUDA 12.1, Python 3.11.8 and R 4.3.3. Training the WDCN model on the full training set took $\sim $2 to 10 min depending on early stop epoch.

## Results

### GWAS results

**Figure 3:**
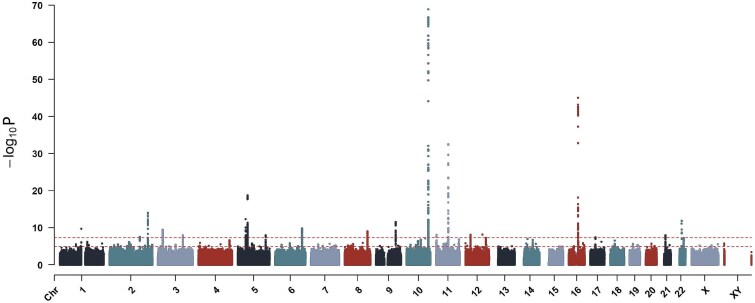
The GWAS results of breast cancer are presented as a Manhattan plot showing the genomic locations (horizontal axis) and $P$ values (vertical axis) of all SNPs passing quality control, and two thresholds ($P < 5 \times 10^{-8}$ and $P < 1 \times 10^{-5}$) are shown.

We first performed a GWAS analysis and identified 931 SNPs located at 21 loci associated with breast cancer at the significant level $P < 5 \times 10^{-8}$. The results were shown in Fig. 3. The SNP with the most significant $P$-value is 10:123340431_GC_G, located on 10q26.13. This locus has been reported in multiple independent studies [39–41] to be associated with breast cancer. It is considered to be located in the promoter region of gene *FGFR2*, and its variant can regulate the expression of *FGFR2* [42]. *FGFR2* is a widely reported gene involved in various types of cancers [43], including breast cancer [44].

Another locus closely associated with breast cancer that we identified is rs4442975, located on 2q35. This region has been reported to be associated with breast cancer [45], particularly triple-negative breast cancer. Evidence suggests that this locus may lie within the enhancer regulatory elements of the *IGFBP5*, which regulates *IGFBP5* expression and increases the risk of breast cancer [45, 46].

**Figure 4:**
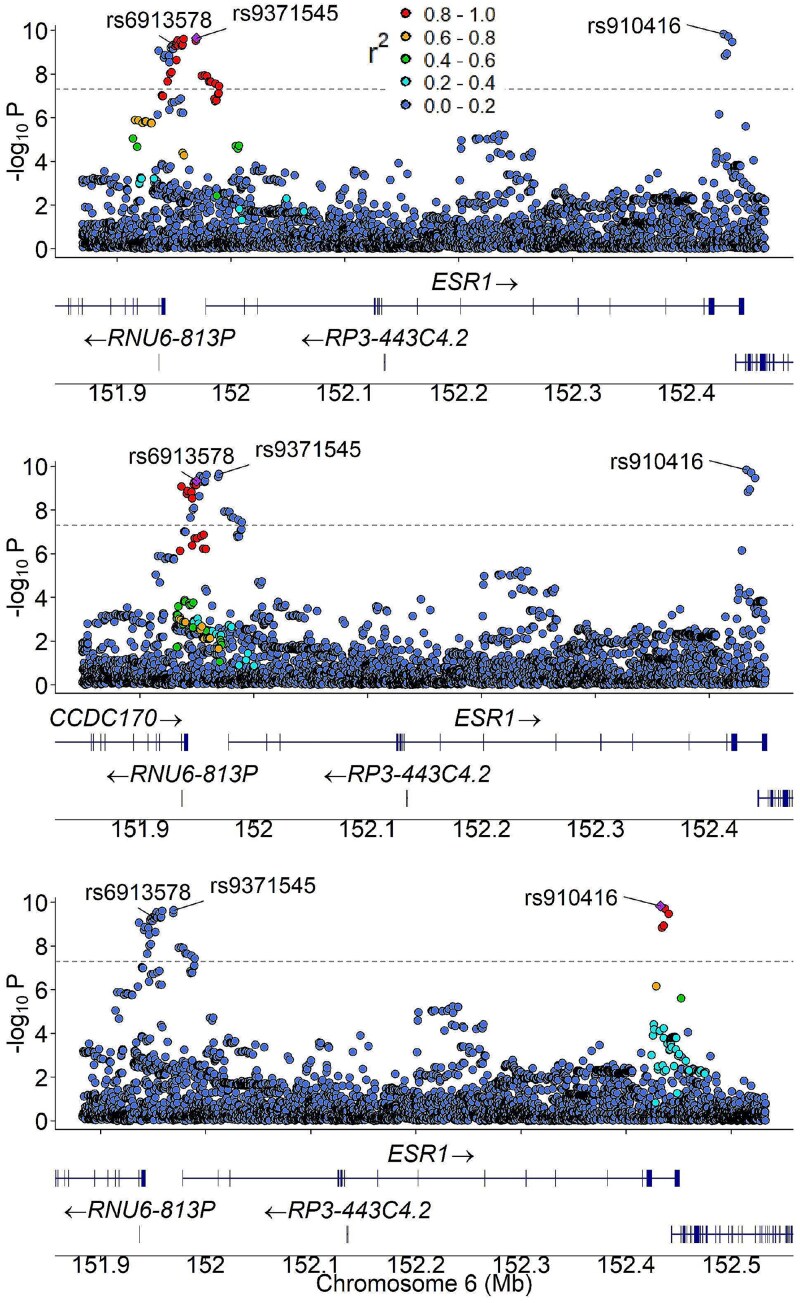
Three independent SNP signals in the 6q25.3 region, where subfigures A, B and C display the LD patterns of rs9371545, rs6913578, and rs910416, respectively, with the index SNPs highlighted by purple diamonds and other SNPs colored according to their LD strength with the index SNP.

In the analysis of each region, we found that there is more than one independent signal per region. For example, in region 6q25.3, we identified three independent signals, as shown in Fig. 4. LD analysis indicated that although rs6913578 and rs9371545 are physically close ( 19.9 K base pair), they reside in different LD blocks: the variants even in low LD ($r^{2}> 0.2$) with each SNP do not overlap. rs6913578 has been previously reported to be associated with breast cancer [47, 48]; some studies suggest a strong association between rs6913578 and rs2046210, with rs2046210 more broadly reported to be associated with breast cancer [49]. In our study, rs6913578 was indeed in strong LD with rs2046210 ($r^{2} = 0.858$), but the $P$-value for rs2046210 ($P = 2.03 \times 10^{-7}$) did not exceed the threshold of $5.0 \times 10^{-8}$. rs9371545 has been reported primarily in association with male breast cancer [50], and shows a modest association in female breast cancer [51], though its effect is less pronounced in the female population. rs910416 is a previously reported breast cancer-associated SNP [52]. Recent studies have shown that, for breast cancer, particularly hormone receptor positive breast cancer, there are both additive and multiplicative interaction effects with the endocrine disrupting chemical DEHP [53]. These three independent signals suggest that subpopulation genetic structure may exist. In addition, there may also be interaction effects between genetic loci and environmental factors, which simple linear aggregation methods, such as PRS, may not effectively capture this effect.

### Model performance in the existing 286 features

To compare the performance of our model, we used a previously widely adopted SNP set [54] consisting of 313 SNPs. However, in our cohort, only 286, covering 91.4% of reported SNPs, passed quality control after filtering. We therefore first evaluated model performance using these 286 SNPs, with the results shown in Table 2. First, using our filtered features, the PRS model achieves performance comparable to that obtained using all the features. For overall breast cancer, our AUROC reached 0.639, whereas the previous study using all 313 SNPs achieved an AUROC of 0.630 [54]; the two results are very similar. This indicates that, within our selected SNP set, the PRS approach can achieve performance comparable to that obtained using all 313 SNPs. The Random Forest model showed the poorest performance, with an AUROC below 0.6. The classical machine learning models, SVM and XGBoost, demonstrated comparable performance; however, both were inferior to the PRS approach. The AUROC values of PRS, SVM, and XGBoost did not exceed 0.64, whereas all three deep learning models achieved AUROC values greater than 0.64. Among these methods, our proposed model achieved the best performance in terms of AUROC, while the DCN model performed better with respect to recall, F1 score, and accuracy. The PRS model performed better with precision. These findings suggest that deep learning models may capture nonlinear relationships between SNPs and breast cancer risk, leading to improved predictive performance. We also performed Delong's test. Unfortunately, the results showed that our method was not significantly superior to any of the other methods, with the sole exception of Random Forest (Supplementary Table S4). This may be because genetic prediction alone offers limited improvement for breast cancer risk estimation; the marginal gain is simply too small to reach statistical significance.

**Table 2:** Performance on 286 SNPs.

Method	AUROC	Precision	Recall	F1 score	Accuracy
WDCN (ours)	**0.6439**	0.6146	0.5696	0.5913	0.6062
PRS	0.6390	**0.6188**	0.5594	0.5876	0.6074
RF	0.5962	0.5622	0.6451	0.6008	0.5714
XGBoosts	0.6367	0.6099	0.5837	0.5965	0.6052
SVM	0.6378	0.6168	0.5661	0.5904	0.6072
WAD	0.6421	0.6002	0.6311	0.6153	0.6053
DCN	0.6410	0.5975	**0.6732**	**0.6331**	**0.6099**

The highest value for each metric is shown in bold.

Given that non-genetic factors also contribute to breast cancer development, we incorporated several non-genetic features, including body mass index (BMI), alcohol consumption, age, time since menopause, smoking history, and family history of breast and prostate cancer, into our model alongside genetic features. The performance of different models is presented in Table 3. After integrating these factors, the AUROC values of all models increased to above 0.80, indicating that the inclusion of non-genetic information substantially improves breast cancer risk prediction.

**Table 3:** Performance on 286 SNPs with non-gene factors.

Method	AUROC	Precision	Recall	F1 Score	Accuracy
WDCN (ours)	**0.8865**	**0.8688**	0.7109	**0.7820**	**0.8018**
PRS	0.8531	0.7465	0.7806	0.7632	0.7578
RF	0.8407	0.8585	0.6229	0.7220	0.7601
XGBoosts	0.8705	0.8446	0.6788	0.7527	0.7769
SVM	0.8546	0.7377	**0.8040**	0.7694	0.7591
WAD	0.8678	0.8280	0.6984	0.7577	0.7766
DCN	0.8780	0.8348	0.7276	0.7775	0.7918

The highest value for each metric is shown in bold.

Among the evaluated methods, the Random Forest model remained the worst-performing model, with an AUROC slightly above 0.84. The support vector machine (SVM) model showed performance comparable to the conventional PRS approach, with both achieving AUROC values exceeding 0.85. In contrast to the SNP only models, XGBoost demonstrated a more pronounced improvement after incorporating non-genetic factors, outperforming the WAD deep learning model with an AUROC above 0.87, although it remained slightly inferior to the DCN model. Notably, our proposed model was the only approach achieving an AUROC greater than 0.88 and showed superior performance across four evaluation metrics, including AUROC, precision, F1 score, and accuracy. We performed Delong's test, and this time our method significantly outperformed all other methods. Detailed results are shown in Supplementary Table S6. This indicates that by incorporating non-genetic factors, our model can capture more interactions, leading to a greater improvement in performance compared to the other methods.

**Figure 5:**
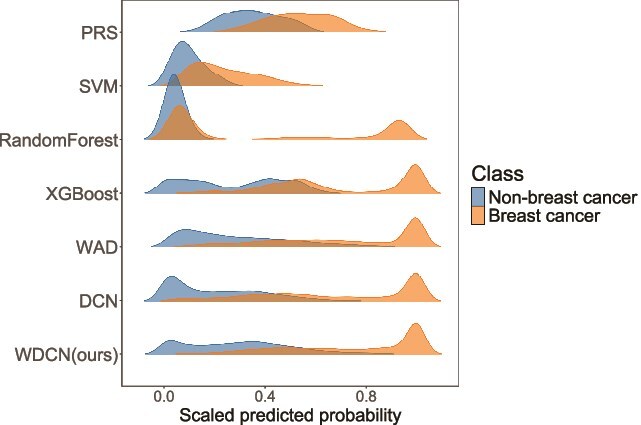
The scaled predicted probability distributions of all seven models, showing the distributions of predicted probabilities for the non-breast cancer and breast cancer cohorts.

To facilitate a fair comparison of model characteristics, we first rescaled the scores or probability produced by all methods to a common range of 0 to 1 in the test data set. The distributions of these standardized values across different population cohorts were then visualized, as shown in Fig. 5. The PRS approach exhibited a unimodal distribution in both breast cancer and non-breast cancer cohorts, resembling a normal distribution. This smooth pattern is likely attributable to the linear nature of the PRS scoring scheme.

The SVM model also demonstrated a unimodal structure; however, the height and spread of the peaks differed noticeably between the two cohorts. In contrast, the Random Forest model showed a unimodal distribution in the non-breast cancer cohort but a bimodal distribution in the breast cancer cohort, with one of the peaks substantially overlapping with the non-breast cancer distribution. This observation helps explain the relatively poor performance of Random Forest, suggesting that a subset of breast cancer individuals were not correctly distinguished from non-breast cancer cohort. Better performing models, such as XGBoost, WAD, DCN, and WDCN, displayed either bimodal distributions or unimodal distributions with long tails in both cohorts. The primary peaks between non-breast cancer cohort and breast cancer cohort showed minimal overlap, whereas the long tails or secondary peaks partially overlapped. This pattern suggests that these models are effective at distinguishing individuals at the either extremes of risk (i.e. very healthy or very high risk individuals), while their discriminative ability for individuals in the intermediate risk range remains comparatively limited.

### Application of traditional SNPs adds independent significant SNPs

Previous GWAS analyses suggested the presence of potential population substructure, and different significant loci may correspond to distinct biological mechanisms underlying breast cancer development. Therefore, we incorporated additional independent genome-wide significant variants identified in the earlier GWAS analysis to evaluate whether expanding the SNP set could further improve model performance. Specifically, 31 independent significant loci were added, resulting in a total of 317 SNPs. These 317 SNPs were then used to re-evaluate both existing models and our proposed approach. All results are presented in Table 4. 

**Table 4:** Model performance on 317 SNPs.

Method	AUROC	Precision	Recall	F1 score	Accuracy
WDCN (ours)	**0.6464**	0.6043	**0.6340**	**0.6188**	**0.6094**
PRS	0.6303	**0.6195**	0.5135	0.5615	0.5990
RF	0.6012	0.6013	0.4307	0.5019	0.5726
XGBoosts	0.6348	0.6098	0.5597	0.5837	0.6008
SVM	0.6257	0.6097	0.5415	0.5736	0.5974
WAD	0.6444	0.6089	0.6062	0.6075	0.6084
DCN	0.6424	0.6056	0.6141	0.6098	0.6071

The highest value for each metric is shown in bold.

The Random Forest model remained the worst-performed method; however, compared with the model using 286 SNPs, its performance improved, with the AUROC exceeding 0.60. In contrast, the PRS, SVM, and XGBoost models showed slight decreases in AUROC. The AUROC values of PRS and XGBoost remained above 0.63, whereas the SVM model decreased to 0.6257.

All three deep learning models (WAD, DCN, and WDCN) demonstrated performance improvements, maintaining AUROC values above 0.64. This finding suggests that deep learning approaches may be better suited to capturing non linear effects such as subtle differences in genetic background and heterogeneous pathogenic mechanisms across subpopulations. Among these models, the PRS approach achieved the highest precision among all evaluated methods. Notably, our WDCN model achieved superior performance in terms of AUROC, recall, F1 score, and accuracy.

**Figure 6:**
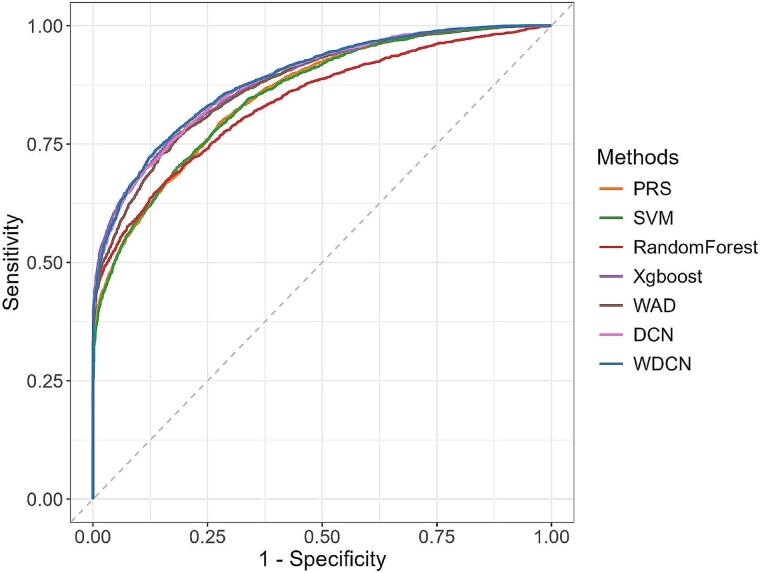
The AUROC curves of all seven methods are shown, with the random forest model demonstrating the poorest performance, SVM and PRS achieving moderate performance, and XGBoost, WAD, DCN, and WDCN showing relatively strong performance with minor differences, among which WDCN achieves the best overall performance.

We subsequently incorporated non-SNP features into the models to further evaluate predictive performance.All results are presented in Table 5. The resulting AUC curves are presented in Fig. 6. The evaluated methods remained broadly grouped into three performance tiers. Random Forest constituted the lowest tier, with an AUROC below 0.84. SVM and PRS formed an intermediate tier, both achieving AUROC values slightly above 0.85; notably, the PRS approach outperformed the model using 286 SNPs combined with non-genetic factors.

The highest performing tier consisted of WDCN, XGBoost, WAD, and DCN, all of which achieved higher AUROC values than the corresponding models based on 286 SNPs with non-genetic factors. In particular, WDCN, DCN, and XGBoost exceeded an AUROC of 0.88. Among all evaluated approaches, our WDCN model consistently demonstrated the best performance in terms of AUROC, precision, and accuracy, whereas PRS achieved superior recall, and XGBoost achieved superior F1 score. In all scenarios, the models that incorporated non-genetics or clinical information performed better than those using only genetic data. This suggests that if more breast cancer-related clinical variables can be identified, the model has the potential to achieve more accurate predictions. Similarly, we conducted Delong's tests, and the detailed results are presented in Supplementary Tables S8 and S10. The trend was consistent with the preceding models: the improvement over other methods was not significant when only genetic factors were used, but became statistically significant after non-genetic factors were incorporated. Among all the models, only the XGBoost model remained not significant even after the addition of non-genetic factors.

For the above models, we also used a non-European ancestry dataset from the UK Biobank as an external validation set. Owing to substantial differences in genetic background across populations, the AUC of all methods declined. Detailed information on the non-European subgroups in the UK Biobank and their classification is provided in Supplementary Table S2, and the validation results for each model are reported in Supplementary Tables S3, S5, S7, and S9. As expected, all models suffered a considerable drop in AUC and other performance metrics on the non-European validation set. Nevertheless, our method still slightly outperformed the other approaches. In some cases, such as on the dataset combining 286 SNPs with non-genetic factors, our method only marginally surpassed DCN and other comparable models.

**Table 5:** Model performance on 317 SNPs with non-genetics factors.

Method	AUROC	Precision	Recall	F1 score	Accuracy
WDCN (ours)	**0.8872**	**0.8547**	0.7209	0.7821	**0.7992**
PRS	0.8549	0.7416	**0.7967**	0.7682	0.7595
RF	0.8378	0.8241	0.6513	0.7276	0.7561
XGBoost	0.8833	0.8227	0.7522	**0.7859**	0.7951
SVM	0.8532	0.7929	0.7001	0.7436	0.7586
WAD	0.8769	0.8310	0.7279	0.7760	0.7899
DCN	0.8841	0.8183	0.7525	0.7840	0.7927

The highest value for each metric is shown in bold.

### Assessment of risk increase

**Figure 7:**
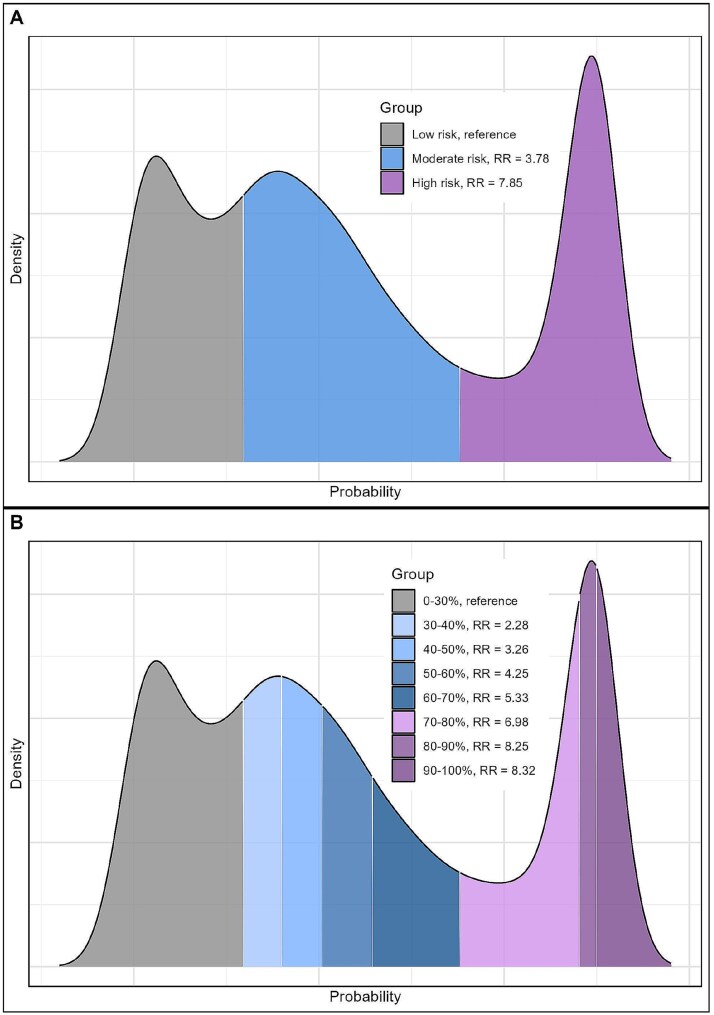
Risk classification, with subfigure A showing three-level risk stratification into low, intermediate, and high-risk groups, and subfigure B presenting a further subdivision into detailed strata based on 10% increments of predicted probability.

To enable more effective risk stratification, we used the predictions generated by our model based on the 317 SNPs combined with non-genetic factors to perform a rough population stratification (Fig. 7A). Individuals were ranked according to predicted risk probability, with the lowest 30% defined as the low risk group, those between 30% and 70% as the intermediate risk group, and those above 70% as the high risk group.

Consistent with the distribution pattern observed in Fig. 5, the overall distribution of predicted probabilities across all individuals did not follow a standard normal distribution. Instead, the distributions within the non-breast cancer and breast cancer groups exhibited long-tailed patterns or secondary peaks. When the two groups were combined, the overall distribution formed a trimodal pattern, with each peak largely corresponding to the low, intermediate, and high risk strata.

We then compared the relative risk (RR) of breast cancer in the intermediate and high risk groups with that of the low-risk group. The results showed that individuals in the intermediate risk group had approximately a three-fold increased risk (3.78 95% CI: 3.34–4.28), while those in the high risk group exhibited nearly an approximate seven-fold higher risk (7.85, 95% CI: 6.98–8.83) compared with the low risk population.

For a more detailed evaluation of risk elevation, the intermediate and high risk populations were subdivided into different strata, with each 10% increase in predicted probability defining an additional risk category (Fig. 7B). We calculated the RR increase for each subgroup compared with the reference group.

Within the intermediate risk group, subgroups 30%–40%, 40%–50 %, 50%–60%, and 60%–70% exhibited RR of 2.28 (95% CI: 1.92 = 2.70), 3.26 (95% CI: 2.81–3.79), 4.25 (95% CI: 3.70–4.88) and 5.33 (95% CI: 4.68–6.08), respectively, compared with the low risk reference group, whereas in the high risk subgroup, the corresponding RRs were 6.98 (95% CI: 6.18–7.89), 8.25 (95% CI: 7.34–9.28), and 8.32 (95% CI: 7.04–9.36). Broadly, for every 10% increase in predicted probability, the RR increased by one-fold, ranging from 2.28-fold in the lowest subgroup to 8.32-fold in the highest. This risk stratification approach further confirms that our model can effectively differentiate individuals with varying levels of breast cancer risk.

### Feature importance analysis

**Figure 8:**
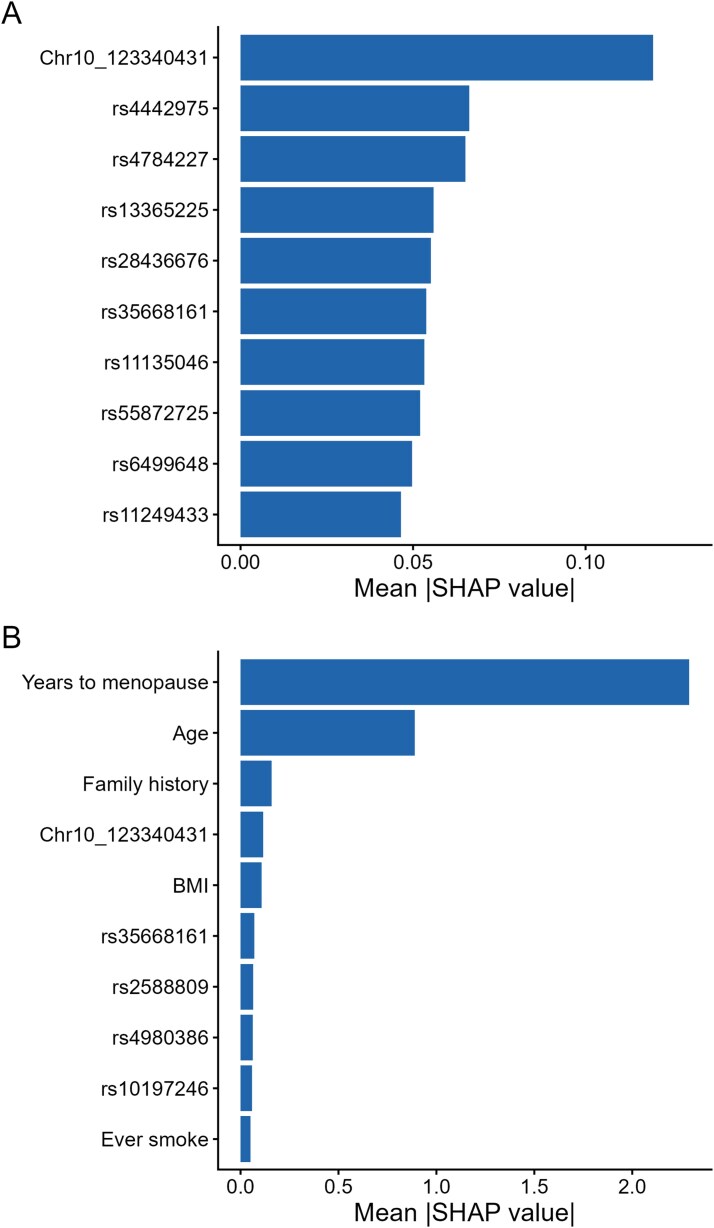
Feature importance analysis, with subfigure A showing the ranking and SHAP values of the top 10 most important features in the WDCN model using 317 SNPs, and subfigure B presenting the corresponding analysis for the WDCN model integrating 317 SNPs with non-genetic factors.

We employed SHAP to analyze feature importance for the WDCN method under two feature sets: one using only the 317 SNP features, and the other using the 317 SNP features plus an additional 6 non-genetic features. This allowed us to know the contribution of individual features to the model’s predictions. Figure 8 presented the top ten most important features identified by each of the two models. Chr10_123340431 was the top-ranked feature among the 317 SNPs. After adding 6 non-genetic features, it remained the top SNP, highlighting its importance for breast cancer risk assessment. Years to menopause was the most important feature overall, followed by age. Despite their similarity, these two factors may reflect distinct pathogenic mechanisms. We also observed that the importance ranking of some SNPs changed dramatically after including non-genetic features. For instance, rs2588809 rose from 108th to 7th, prompting us to explore the reasons behind this shift.

### Identification of interaction features

**Figure 9:**
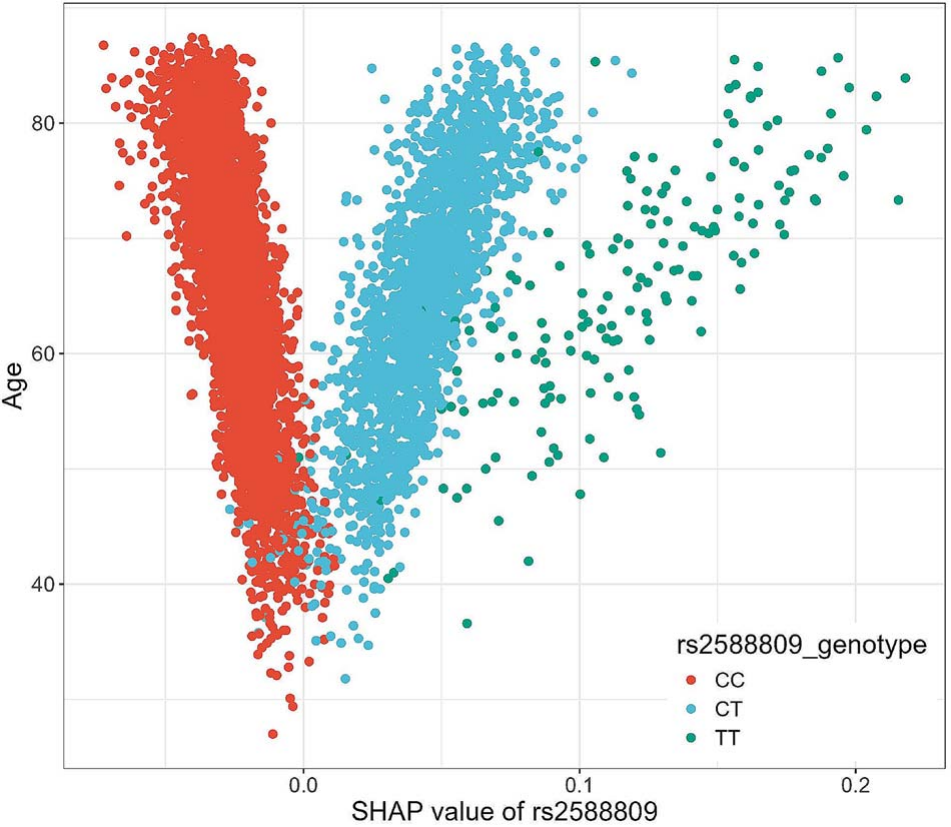
Interaction analysis between rs2588809 and age, illustrating the variation of SHAP values for rs2588809 across different genotypes.

Computing all pairwise interactions among 321 features would require 102 720 calculations, demanding substantial time and computational resources. Since most of these combinations are unlikely to be meaningful, exhaustive calculation is not a wise strategy. Our previous results showed that adding non-genetic features substantially altered the importance of rs2588809. Although this change is not necessarily due to interaction effects, we chose to explore the possibility. Therefore, we only tested the interactions between rs2588809 and each of the 6 non-genetic features. Fortunately, we detected an interaction between rs2588809 and age, as presented in Fig. 9.

For rs2588809, the CC genotype yields negative SHAP values that are inversely proportional to age, indicating a protective effect against breast cancer. The CT genotype yields positive SHAP values proportional to age, and the TT genotype yields consistently positive SHAP values also proportional to age, both indicating susceptibility to breast cancer. Showing opposite trends across different genotypes is exactly the sign of an interaction. This figure demonstrates that rs2588809 interacts with age in a genotype-dependent manner, nonlinearly modulating breast cancer risk.

rs2588809 has long been found to significantly influence breast cancer risk [55]. It is located in an intronic region of gene *RAD51B*. The *RAD51B* plays a key role in the homologous recombination pathway of DNA damage repair. Moreover, age affects the accumulation of somatic mutations, and in combination with the rs2588809 variation, may nonlinearly accelerate the development of cancers, including breast cancer.

## Discussion

In this study, we first performed a preliminary GWAS analysis on breast cancer cases from the UK Biobank, validating several SNPs previously associated with breast cancer risk. During the analysis, we observed potential substructure within the population genetic background. To account for this complexity and to capture relationships confounded by both subpopulation structure and heterogeneous disease mechanisms, we developed the wide deep and cross network (WDCN) method. This approach was evaluated across four feature space: two containing only SNPs and two incorporating both SNPs and non-genetic factors. Our results demonstrated that the WDCN method outperformed PRS, classical machine learning models, and comparable deep learning approaches. Using SNPs alone, the AUROC values reached 0.6439 and 0.6464. Incorporating non-genetic factors further improved the AUROC to 0.8865 and 0.8872. In population stratification analyses, applying our method with comprehensive features, we observed that individuals in the highest risk group (top 30%) had a 7.85-fold increased risk of breast cancer compared with those in the lowest risk group (bottom 30%). These findings confirm the effectiveness of our approach and suggest its potential utility for refined population-level risk stratification and personalized screening strategies. Finally, we performed SHAP analysis and found that years to menopause and Chr10_123340431 were the most important features among non-genetic and genetic factors, respectively. We also conducted interaction analysis and discovered an interaction between rs2588809 and age. This may provide a new perspective on the role of rs2588809 in breast cancer.

SNP datasets for breast cancer risk assessment generally include 313 SNPs like ours, as well as larger sets such as 3820 SNPs, which are mainly used for evaluating breast cancer subtypes, and other self built SNP panels, e.g. 180 SNPs and 5218 SNPs. Using more SNPs can theoretically provide additional information, and combined with better methods, it can, in theory, lead to more accurate results. Mavaddat et al. [54] used 77, the same 313 as ours, and 3820 SNPs to calculate breast cancer risk. Across all breast cancer cases, the AUROCs reached 0.603, 0.630, and 0.636, respectively. For estrogen receptor positive (ER-positive) subtype breast cancer, all three models performed best, reaching 0.615, 0.641, and 0.647, respectively [54]. For overall risk assessment, the 3820 SNP model did not outperform our method. However, since the UKBB does not provide detailed breast cancer subtype information [56], our method cannot be compared with theirs in terms of subtype accuracy. Yiangou et al. used a cohort of fewer than 1000 individuals from Crate, employing 313 SNPs to assess breast cancer risk, and ultimately achieved an AUC of 0.653 [57]. The higher results may be due to the specific characteristics of the population and the bias introduced by the relatively small sample size used. Evans used 313 SNPs and incorporated information such as breast density, achieving an AUC of 0.665 [58]. This is slightly higher than our results without any clinical information, but far lower than our model that includes more comprehensive information. For those using custom-built SNP datasets, Shieh et al. used the PRS method with 180 SNPs to estimate breast cancer risk in European and Latin American populations, obtaining similar results in both groups, with an AUROC of $\sim $0.63 [59]. Khera et al. used 5218 SNPs in UKBB dataset and achieved an AUC of 0.68 [19]. For the studies that use deep learning based models, Peng et al. developed a deep learning based method called DeepRisk, using 3,830 SNPs, and achieved an AUC of 0.6227 [28]. Its method performed worse than ours, even when using more SNPs. In summary, our method shows advantages over traditional approaches when using the same number of SNPs. Even with more SNPs, our method still offers certain benefits and outperforms other methods, including those based on deep learning. Furthermore, incorporating additional clinical information further improves the predictive accuracy of our method.

Our method also has certain limitations, especially when considering variants that have large effects but are very rare. *BRCA1* and *BRCA2* are well-known key genes in breast cancer research. However, in our dataset, information on these critical genotyping sites was lacking. This is likely because *BRCA1* and *BRCA2* mutations are rare, and the UK Biobank cohort consists predominantly of older individuals, whereas breast cancers driven by *BRCA1* and *BRCA2* mutations tend to occur at earlier ages. Since carriers of these mutations often have a family history of breast cancer, including family history information in our model partially compensates for this missing data. Moreover, breast cancer risk is influenced by genes beyond *BRCA1* and *BRCA2*. Therefore, we also incorporated independent genome-wide significant loci identified in our GWAS analysis, which theoretically makes our model more comprehensive. Consistently, this inclusion led to measurable improvements in model performance.

Another limitation is the improvement in the performance of the model using only genetic factors. Although our method appeared to outperform other methods in both models using only genetic factors, the Delong's test indicated that this difference did not reach statistical significance. This may be because the predictive power of genetic factors is already near saturation, and the minor improvement was not statistically significant.

The final limitation concerns generalizability to other ethnic groups. Because data on other ancestries are scarce and difficult to obtain, our method performs well only in UKBB European populations, with reduced effectiveness elsewhere. This underscores the need to expand research to other ethnic groups.

Traditional and widely used models such as Tyrer-Cuzick and Gail are based on population epidemiological survey data. They select non-genetic factors and risks associated with breast cancer and calculate an individual's disease probability through a multiplicative approach. Because UK Biobank cannot provide or cannot accurately provide factors such as biopsy history, atypical hyperplasia, family history of ovarian cancer, and family history of male breast cancer, it is not feasible to directly compare the performance of our method with these two traditional models. However, indirect comparisons can be made via the relevant literature. The Gail model typically yields an AUC of 0.58–0.63 [60, 61], while Tyrer-Cuzick can reach 0.63–0.69 [62, 63]. Given that our method achieves an AUC above 0.8, our approach clearly provides more accurate stratification in terms of discriminating affected individuals. Nevertheless, the Gail and Tyrer-Cuzick models do not rely on genetic sequencing data and can be computed simply by filling in a questionnaire. They are superior to our method in terms of clinical convenience and resource utilization. At the same time, this also highlights the strengths of our model: our model is able to capture interactions among features, offers better discrimination, and has the potential to uncover pathogenic mechanisms of breast cancer, thereby providing additional value for screening, prevention, and even treatment.

Regarding computational resources and time, PRS is indeed the method that consumes the least computational resources and achieves the fastest speed. Although our method requires slightly more resources, this increase is still acceptable. In practice, when training a model on a GPU with 24 GB of memory, resource usage never exceeds 15% of the total available. Moreover, a single model training session takes no more than 10 min. These computational demands can also be met by a moderately high specification personal computer in real-world applications. This greatly enhances the potential of our model for clinical deployment.

Our model outperformed the linear weighted PRS approach, suggesting that nonlinear relationships or interactions exist between genes, as well as between genes and environmental factors. We have only found one gene-environment interaction, which is far from enough; more interactions should be uncovered. Such interactions could further refine population stratification. In future studies, we aim to identify specific interactions to enable more granular risk stratification and to provide tailored screening and clinical guidance based on the characteristics of distinct population subgroups.

**Key Points**

- A novel deep learning model WDCN for breast cancer risk prediction was developed and demonstrated superior performance to PRS and other benchmark methods across four feature sets in the UK Biobank dataset.
- Our findings suggest that distinct population substructures may exist within the UK Biobank breast cancer cohort, further highlighting the potential advantages of WDCN.
- This WDCN demonstrated strong breast cancer risk stratification in individuals of European ancestry. After ranking individuals by predicted risk, those in the highest risk 30% exhibited an approximately eight-fold greater risk of developing breast cancer compared with those in the lowest risk 30%. This provides a reference for tailoring screening recommendations to different risk groups.
- An interaction between rs2588809 and age was identified in breast cancer risk, providing new insights into disease mechanisms and supporting personalized health recommendations based on genetic profile and age.

## Supplementary Material

sup_bbag412

## Data Availability

The example of WDCN is publicly available at: https://github.com/xuhanshi/WDCN/tree/main.
