# Supplementary material for: WDCN: a comprehensive neural network based approach for estimating breast cancer risk
Source: Brief Bioinform. 2026 Aug 6;27(4):bbag412. doi: 10.1093/bib/bbag412 (PMC13446515; doi:10.1093/bib/bbag412)
Supplement: Supplementary file 1 [file sup_bbag412.pdf]

WDCN: a comprehensive neural network based approach for estimating breast cancer risk

Hanshi Xu[1], Guangquan Zhang[1], Hua Lin[2], Mark Grosser[2], and Jie Lu*[1]

[1]Australian AI Institute, Faculty of Engineering and Information Technology, University of Technology Sydney, 61 Broadway, Sydney, NSW 2007, Australia
[2]23Strands, 26-32 Pirrama Road, Pyrmont, NSW 2009, Australia

*Corresponding author: jie.lu@uts.edu.au

Supplementary Table S 1: Hyperparameter tuning analysis.

Parameter name	Value range	Model 1*	Model 2 *	Model 3 *	Model 4 *
Batch size	1024, 2048, 4096	2048	2048	2048	2048
Learning rate	0.05, 0.01, 0.005, 0.001, 0.0001	0.001	0.001	0.001	0.001
Cross layers	3 ~12	3	11	3	6
Deep layers	3 ~5	4	3	3	3
Deep layers units	[256,64,16,4], [128,32,8], [128,16,2], [256,128,64,32,16], [512,128,32,8,2]	[128,32,8]	[256,64,16,4]	[128,16,2]	[128,16,2]
Weight for loss function	10, 11, 12~14(step 0.1), 15,16	12.9	12.9	12.9	12.9
Number of experts	3 ~25(step 1)	18	5	5	12
Low rank	20 ~70 (step 1)	69	67	54	55
Drop rate	0.1 ~0.3(step 0.1)	0.3	0.3	0.2	0.2
Max epoch & 50,100,200	50	50	50	50	
Weight decay	10^{-4}	10^{-4}	10^{-4}	10^{-4}	10^{-4}
Patience for early stop	15	15	15	15	15
Min improvement to reset patience	10^{-4}	10^{-4}	10^{-4}	10^{-4}	
Learning rate scheduler reduction factor	0.5	0.5	0.5	0.5	0.5
Learning rate scheduler patience	5	5	5	5	5

Model 1 represents the 286 SNPs model.
Model 2 represents the 286 SNPs with non-genetics factors model.
Model 3 represents the 317 SNPs model.
Model 4 represents the 317 SNPs with non-genetics factors model.

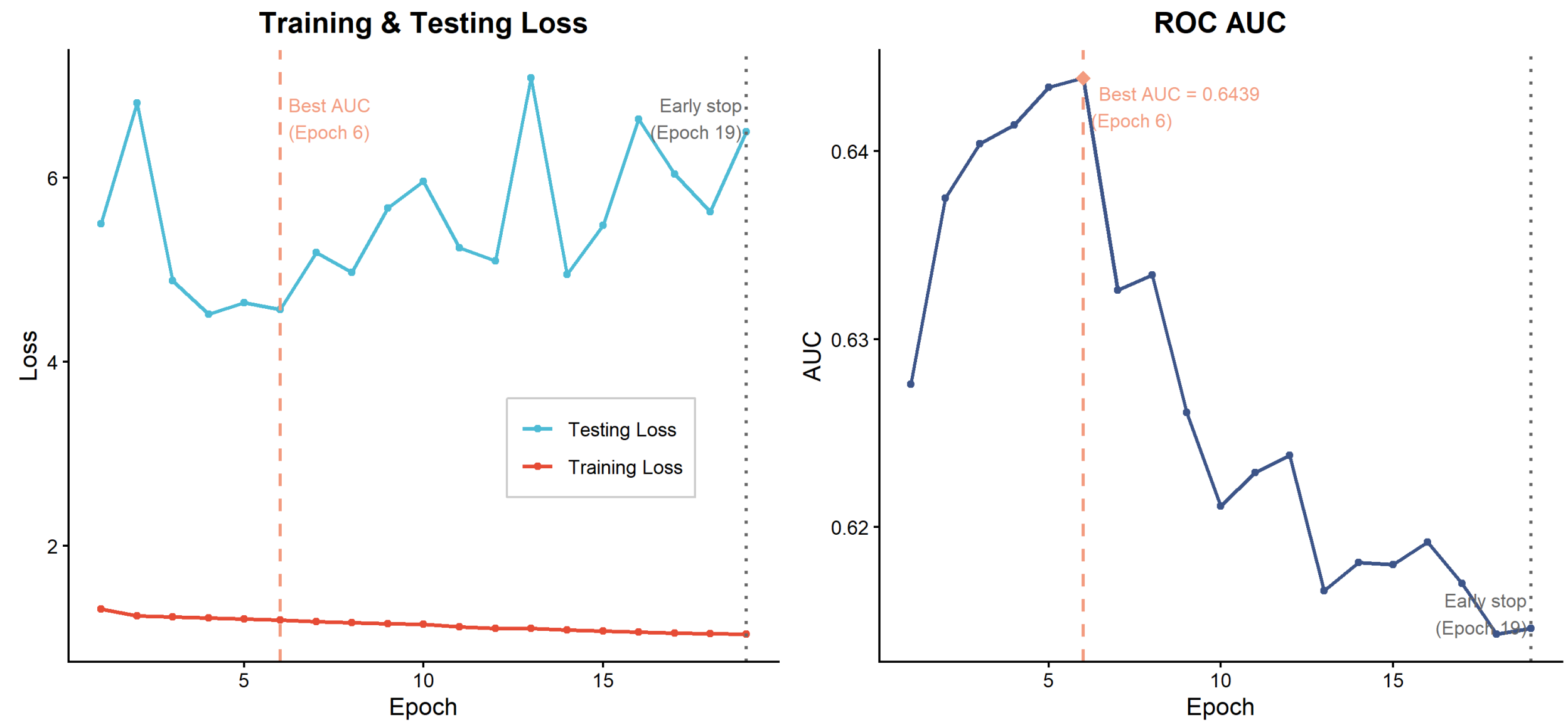

Supplemental Figure S 1: **The loss decrease and AUC change of the 286 SNPs model.** This figure shows how the loss and AUC change with epochs.

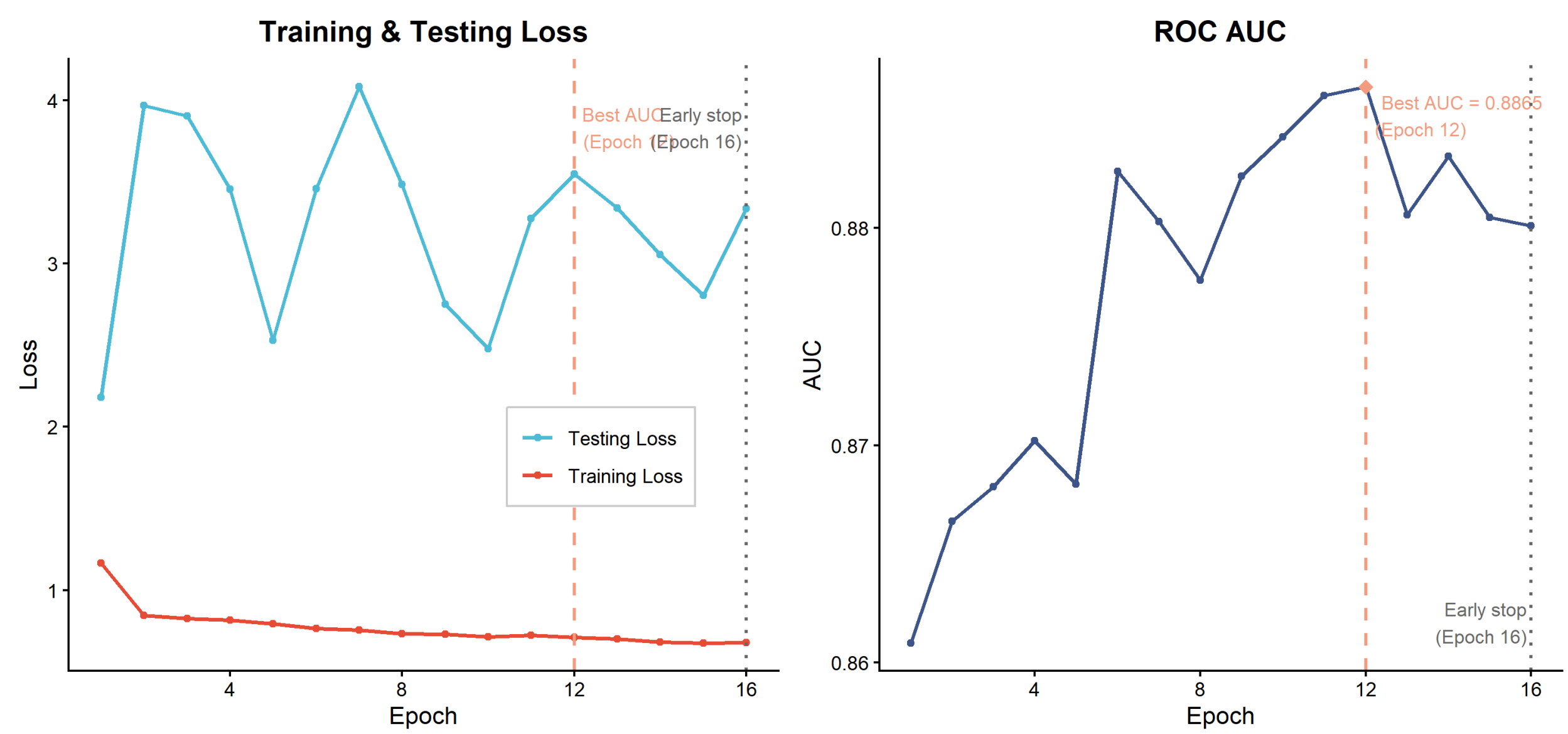

Supplemental Figure S 2: **The loss decrease and AUC change of the 286 SNPs with non-genetics factors model.**

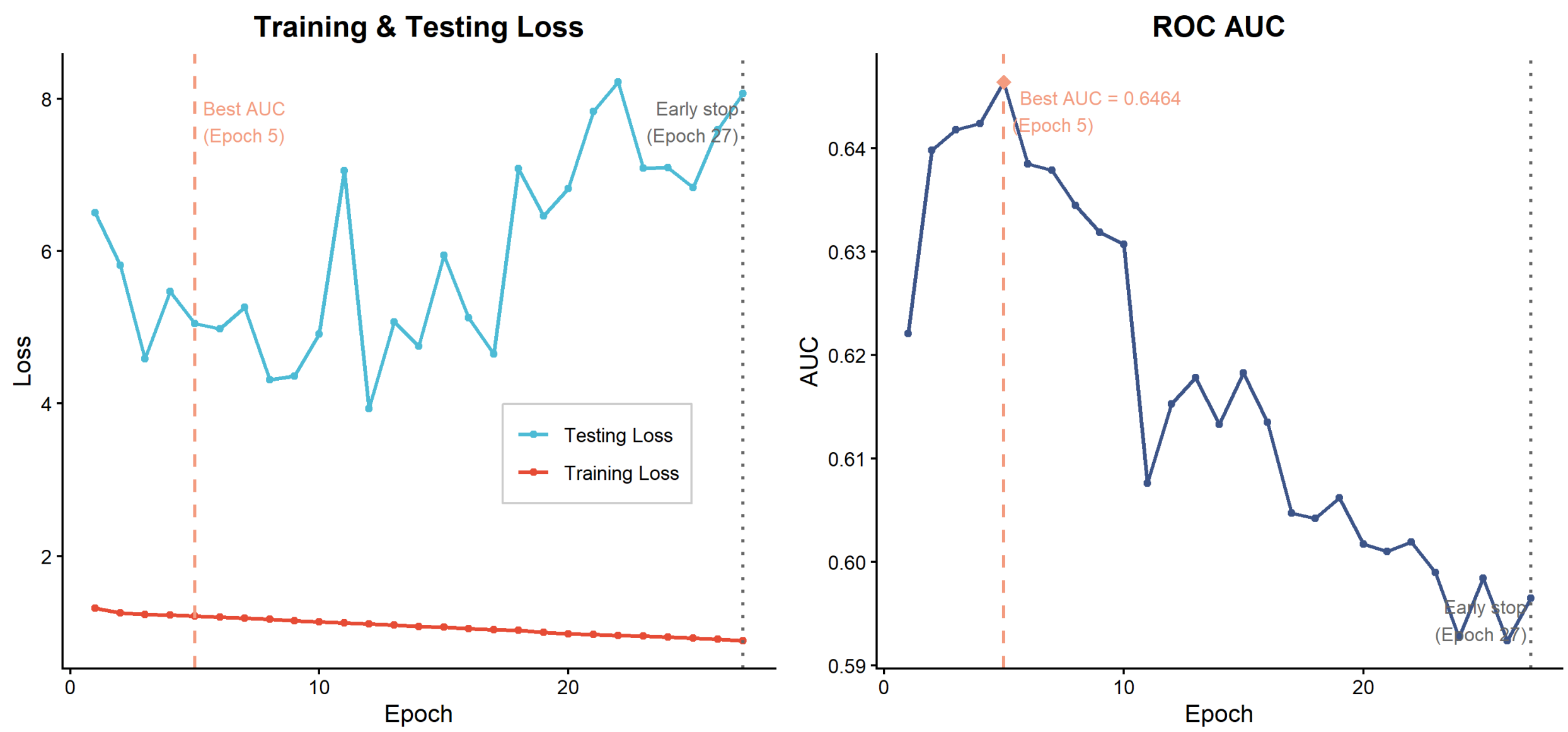

Supplemental Figure S 3: **The loss decrease and AUC change of the 317 SNPs model.**

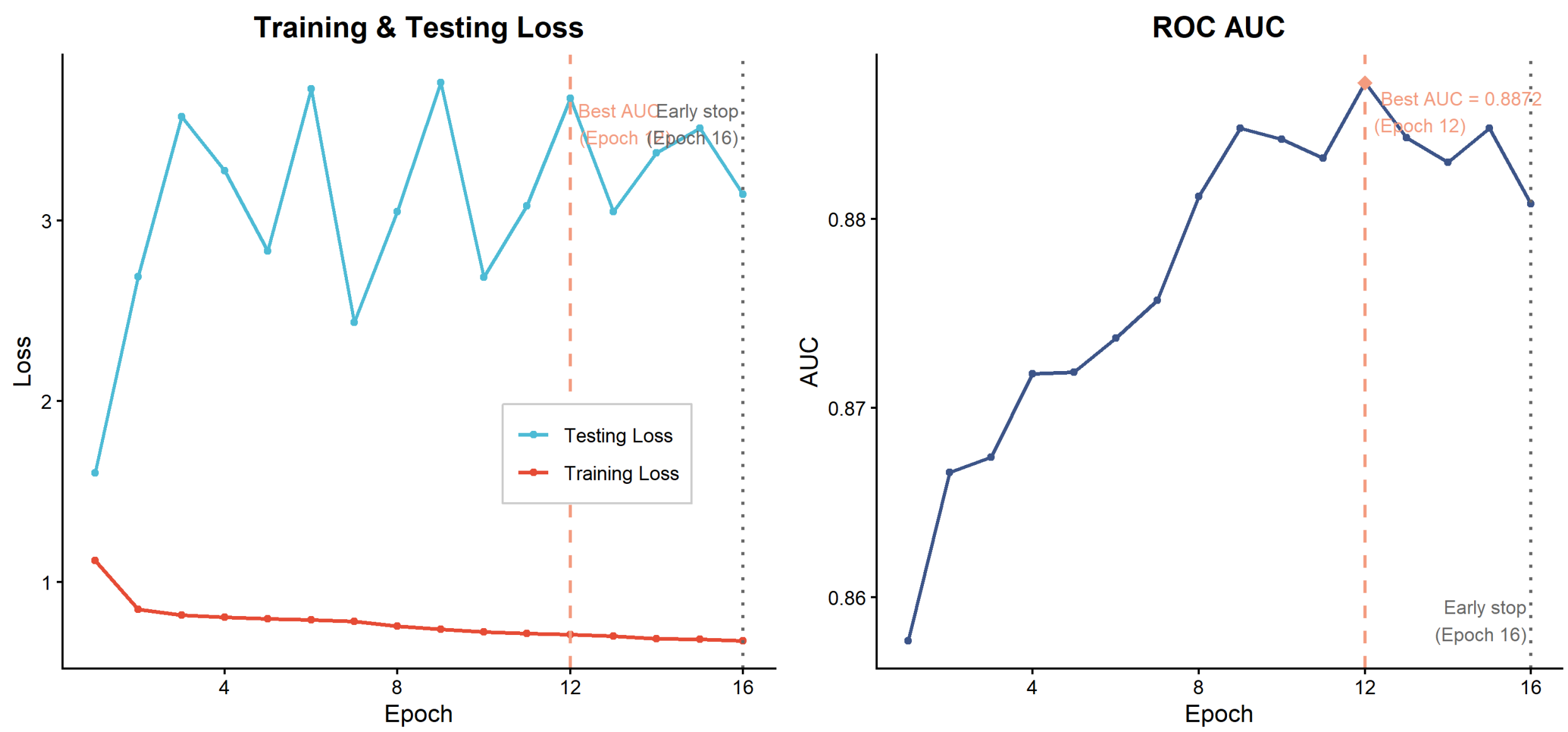

Supplemental Figure S 4: **The loss decrease and AUC change of the 317 SNPs with non-genetics factors model.**

Supplementary Table S 2: Summary of other ethnic groups.

Ethnic groups	Category	Number
African	Breast cancer	51
African	Non-breast cancer	1371
Chinese	Breast cancer	2
Chinese	Non-breast cancer	82
Latin America	Breast cancer	103
Latin America	Non-breast cancer	2190
Mixed	Breast cancer	92
Mixed	Non-breast cancer	1440
Other	Breast cancer	101
Other	Non-breast cancer	1645
Other Asia	Breast cancer	22
Other Asia	Non-breast cancer	481
South Asia	Breast cancer	168
South Asia	Non-breast cancer	2927
Total	Breast cancer	539
Total	Non-breast cancer	10140

Supplementary Table S 3: 286 SNPs model validate on other ethnic groups

Method	AUROC	Precision	Recall	F1 Score	Accuracy
WDCN (Ours)	**0.6118**	0.0616	0.6051	0.1118	0.5859
PRS	0.6021	0.0642	0.6364	0.1167	0.5149
RF	0.5438	0.0583	0.5770	0.1058	0.5078
XGBoost	0.6367	0.0582	0.5176	0.1175	**0.6077**
SVM	0.6087	0.0654	0.6475	**0.1187**	0.5149
WAD	0.5985	0.0602	**0.7254**	0.1111	0.4142
DCN	0.6038	**0.0668**	0.6051	0.1118	0.5859

Supplementary Table S 4: DeLong's test for the 286 SNPs model on the test dataset.

Comparison of different methods	P value
PRS vs WDCN	0.658
SVM vs WDCN	0.562
Random Forest vs WDCN	3.94×10^{-15}
XGBoost vs WDCN	0.489
WAD vs WDCN	0.600
DCN vs WDCN	0.128

Supplementary Table S 5: 286 SNPs with non-genetics features model validate on other ethnic groups

Method	AUROC	Precision	Recall	F1 Score	Accuracy
WDCN (Ours)	**0.8550**	0.0882	0.8738	0.1602	0.5371
PRS	0.8300	0.0876	0.8683	0.1575	0.5371
RF	0.8221	0.0856	0.8479	0.1555	0.5351
XGBoost	0.8550	0.0888	**0.8794**	0.1613	0.5383
SVM	0.8339	0.0867	0.8590	0.1575	0.5362
WAD	0.8543	0.0998	0.8590	0.1789	0.6019
DCN	0.8540	**0.1190**	0.8182	**0.2078**	**0.6851**

Supplementary Table S 6: DeLong's test for the 286 SNPs with non-genetics model on the test dataset.

Comparison of different methods	P value
PRS vs WDCN	2.15×10^{-8}
SVM vs WDCN	8.96×10^{-8}
Random Forest vs WDCN	$< 2.20 \times 10^{-16}$
XGBoost vs WDCN	6.67×10^{-3}
WAD vs WDCN	$< 2.20 \times 10^{-16}$
DCN vs WDCN	3.50×10^{-7}

Supplementary Table S 7: 317 SNPs model validate on other ethnic groups

Method	AUROC	Precision	Recall	F1 Score	Accuracy
WDCN (Ours)	**0.6049**	**0.0668**	0.6436	**0.1210**	**0.5934**
PRS	0.5771	0.0618	0.6122	0.1123	0.5113
RF	0.5331	0.0549	0.5436	0.0997	0.5044
XGBoost	0.5830	0.0591	0.6660	0.1086	0.4484
SVM	0.5990	0.0633	0.6271	0.1150	0.5128
WAD	0.5926	0.0595	0.7106	0.1098	0.4184
DCN	0.5849	0.0582	**0.7570**	0.1080	0.3691

Supplementary Table S 8: DeLong's test for the 371 SNPs model on the test dataset.

Comparison of different methods	P value
PRS vs WDCN	0.122
SVM vs WDCN	0.0415
Random Forest vs WDCN	9.44×10^{-13}
XGBoost vs WDCN	0.284
WAD vs WDCN	0.716
DCN vs WDCN	0.103

Supplementary Table S 9: 317 SNPs with non-genetics factors model validate on other ethnic groups

Method	AUROC	Precision	Recall	F1 Score	Accuracy
WDCN (Ours)	**0.8764**	0.1435	0.7978	0.2432	0.7494
PRS	0.8259	0.0869	0.8609	0.1579	0.5364
RF	0.8146	0.0841	**0.8330**	0.1528	0.5336
XGBoost	0.8568	0.1090	0.8312	0.1927	0.6486
SVM	0.8291	0.0863	0.8553	0.1569	0.5359
WAD	0.8457	0.097	0.8534	0.1742	0.5917
DCN	0.8701	**0.1456**	0.8108	**0.2469**	**0.7504**

Supplementary Table S 10: DeLong's test for the 371 SNPs with non-genetics factors model on the test dataset.

Comparison of different methods	P value
PRS vs WDCN	3.03×10^{-8}
SVM vs WDCN	6.57×10^{-9}
Random Forest vs WDCN	$< 2.20 \times 10^{-16}$
XGBoost vs WDCN	0.483
WAD vs WDCN	7.64×10^{-10}
DCN vs WDCN	0.013
